# IKZF-associated inborn errors of immunity

**DOI:** 10.70962/jhi.20250063

**Published:** 2025-07-18

**Authors:** Motoi Yamashita, Tomohiro Morio

**Affiliations:** 1 Department of Pediatrics and Developmental Biology, Graduate School of Medical and Dental Sciences, Institute of Science Tokyo, Tokyo, Japan; 2 Laboratory for Transcriptional Regulation, RIKEN Center for Integrative Medical Sciences, Yokohama, Japan; 3 Laboratory of Immunology and Molecular Medicine, Advanced Research Initiative, Institute of Science Tokyo, Tokyo, Japan

## Abstract

IKAROS, HELIOS, and AIOLOS are transcription factors predominantly expressed in hematopoietic cells, where they form heteromeric and homodimeric complexes and facilitate transcriptional regulation. IKZF proteins also associate with non-IKZF family proteins, which vary between different immune cell subtypes and their differentiation stages. Heterozygous germline loss-of-function variants in *IKZF1*, *IKZF2*, and *IKZF3* cause IKAROS, HELIOS, and AIOLOS deficiencies, respectively, leading to inborn errors of immunity (IEI). Heterozygous gain-of-function (GOF) variants in *IKZF1* result in IKAROS-GOF disease, characterized by autoimmune and allergic manifestations, whereas dominant-negative IKAROS and AIOLOS variants are associated with combined immunodeficiency. Importantly, patients with IKZF-associated IEI exhibit varying degrees of immunodeficiency, immune dysregulation, and occasional malignancies, and so, disease manifestations differ significantly among the variant types. Therefore, each variant often causes phenotypic heterogeneity, which possibly stems from diverse protein complexes formed by IKZF proteins. Besides immunoglobulin supplementation for patients with B cell defects and hematopoietic cell transplantation for severe cases, molecularly targeted therapies have been investigated for treating IKAROS-GOF disease.

## Teaser

Germline variants in IKZF family proteins cause inborn errors of immunity characterized by variable degrees of immunodeficiency, immune dysregulation, and occasional malignancies.

## IKAROS zinc finger (IKZF) transcription factors and hematopoiesis: Implication of their roles from the mouse studies

IKZF transcription factors belong to the Krüppel-like family of zinc finger (ZF) proteins and play essential roles in multiple stages of hematopoiesis (1). Ikaros, the founding member of this family, was initially identified as a master regulator of lymphocyte development (2, 3, 4). Its homologs Helios, Aiolos, Eos, and Pegasus were later discovered to be Ikaros-binding partners (5, 6, 7, 8). Structurally, IKZF proteins share three or four N-terminal ZFs responsible for DNA binding and two C-terminal ZFs that mediate dimerization. Through these C-terminal ZFs, IKZF family members form homo- or heterodimers, assembling into larger transcriptional complexes, such as Mi-2/nucleosome remodeling deacetylase (NuRD) and Polycomb repressive complex 2 complexes. Together with IKZF proteins, these complexes orchestrate gene regulatory networks that are critical for hematopoietic cell differentiation and function (1, 9).

Mice lacking Ikaros, either through dominant-negative (DN) or null mutations, exhibit severe defects in lymphocyte development, highlighting the role of Ikaros in the development of all lymphoid lineages (2, 4). Ikaros-deficient mice also show reduced long-term repopulating activity of hematopoietic stem cells (HSCs) along with a marked decrease in common lymphoid progenitors (CLPs) (10, 11). In mature lymphocytes, Ikaros plays an essential role in maintaining self-tolerance. Conditional knockout of Ikaros in B cells and regulatory T (Treg) cell–specific deletion of exon 5 of *Ikzf1*, which encodes ZF2 and ZF3, which are known to mediate interactions with Foxp3, disrupts self-tolerance and leads to severe autoimmunity (10, 11).

In contrast to Ikaros, the loss of Aiolos has only a mild effect on overall lymphocyte development (12). However, Aiolos-deficient mice exhibit a breakdown in B cell tolerance, spontaneous autoantibody production, and the development of autoimmunity. Aiolos contributes to T helper (Th) 17 differentiation by suppressing IL-2 expression (13). Helios and Eos contribute to the stability and suppressive function of Treg cells, respectively. Helios-deficient mice do not display overt immunological abnormalities under steady-state conditions; however, older mice develop autoimmune features, including autoantibody production and dysregulated germinal center reactions (14, 15, 16). Moreover, Treg-specific Helios knockout results in autoimmunity and reduced Treg viability, highlighting the essential role of Helios in Treg cell development and function. Similarly, the conditional deletion of Eos in Tregs results in severe autoimmunity and impaired suppressive capacity of Tregs (17), further highlighting the importance of these IKZF molecules in maintaining self-tolerance. Pegasus, encoded by the *IKZF5* gene, is the most evolutionarily divergent member of the IKZF transcription factor family (18); however, its role in hematopoiesis remains poorly understood. However, heterozygous germline loss-of-function (LOF) variants of *IKZF5* have been identified in patients with hereditary thrombocytopenia (19).

Since 2012, germline variants of IKZF transcription factors have been identified in pediatric and adult patients presenting with a wide spectrum of immune abnormalities. To date, *IKZF1*, *IKZF2*, and *IKZF3* have been recognized as causative genes for inborn errors of immunity (IEI) among IKZF members. Their pleiotropic roles, dynamic expression changes during hematopoietic cell differentiation, and functional complexity, potentially mediated by distinct protein complex formations, contribute to the diverse clinical phenotypes and immunological abnormalities observed in IKZF-associated IEI. In patients with distinct IKZF-associated IEI, pathogenic variants, which similarly disrupt the functions of IKZF family of proteins, can equivalently impair immune cell development and function, resulting in overlapping immunological phenotypes. Therefore, it is important to understand IKZF-associated IEI as a unified disease entity, rather than considering each condition in isolation. In this review, we summarize the molecular pathogenesis, clinical manifestations, immunological abnormalities, therapeutic approaches, and outcomes of IKZF-associated IEI.

## IKAROS deficiency

The heterozygous missense variant Y210C in the *IKZF1* gene was first detected in a premature neonate presenting with pancytopenia (20). Following the initial report, heterozygous LOF variants were subsequently identified in cohorts with predominant B cell and antibody deficiencies, as well as in patients with pancytopenia and autoimmunity (21, 22). These findings further established *IKZF1* as the causative gene of IEI. Among *IKZF1* LOF variants causing IEI, DN variants have been identified in patients with severe infectious complications, including *Pneumocystis jirovecii* pneumonia (PCP), whereas variants disrupting IKAROS dimerization are associated with a higher incidence of autoimmunity (23, 24).

**Figure 1.**
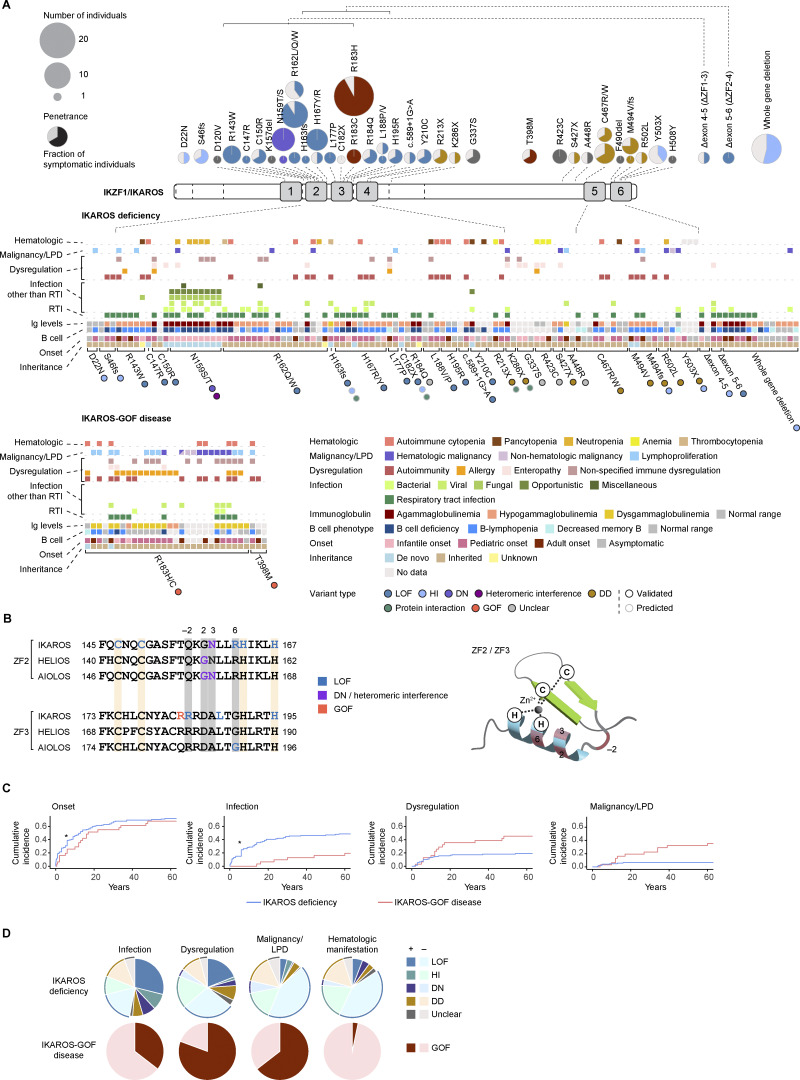
**IKAROS deficiency and IKAROS-GOF disease. (A)** Structure of the IKZF1/IKAROS protein is depicted, highlighting the N-terminal ZFs (ZF1–ZF4) responsible for DNA binding and the C-terminal ZFs (ZF5–ZF6) involved in homo- and heterodimerization, indicated by gray boxes. Exon boundaries within the structure are marked by dotted lines. Above the schematic, disease-causing variants associated with IKAROS deficiency and IKAROS-GOF disease are mapped to their respective positions, along with the number of individuals carrying each variant and the corresponding disease penetrance. Below the structure, the spectrum of clinical phenotypes reported in all known individuals with IKZF1-associated IEI is shown. **(B)** Amino acid sequences of ZF2 and ZF3 from IKAROS, HELIOS, and AIOLOS are shown. Essential amino acids for ZF structure formation are highlighted in orange, and those critical for mediating DNA binding are highlighted in gray. Numbers on both sides indicate amino acid positions within the protein. Positions relative to the start of the alpha helix are marked above the sequence: residues at positions −2, 2, 3, and 6 are critical for DNA binding. Amino acids associated with variants in IEI are indicated in bold. The pathogenic mechanisms of these variants are color-coded. **(C)** Onset of overall disease (first clinical manifestation), infections, immune dysregulation, and hematological malignancies or lymphoproliferation. *: when the onset was described as occurring in childhood without a specific age provided, it was represented as age 6 in the graph. **(D)** Frequencies of symptomatic individuals for each phenotype are summarized. Color codes represent variant types linked to IKAROS deficiency. C, cysteine; H, histidine; LPD, lymphoproliferative disorder.

To date, more than 120 patients and 40 causative variants have been reported in the literature (Fig. 1 A, detailed in this review and [25]) (20, 21, 22, 23, 24, 26, 27, 28, 29, 30, 31, 32, 33, 34, 35, 36, 37, 38, 39, 40, 41, 42, 43, 44). Heterozygous germline *IKZF1* variants have been identified in several whole-exome sequencing studies in patients with IEI (45, 46, 47).

### Genetics and pathogenesis mechanism

IKAROS deficiency is caused by heterozygous germline LOF variants of the *IKZF1* gene. Traditionally, missense variants that impair the DNA binding of IKAROS have been categorized as haploinsufficiency (HI). However, accumulating evidence from IKAROS deficiency and other IKZF-associated IEI suggests that some of these missense variants may contribute to the disease via alternative mechanisms, such as disrupted protein–protein interactions and interference with the function of heteromerizing partner proteins. Therefore, in this review, we distinguish missense LOF variants from HI, defining HI as a complete loss of expression of the pathogenic allele. Most missense variants causing IKAROS deficiency were located in the DNA-binding N-terminal ZFs (ZF1–4) (Fig. 1, A and B). Among the stop-gain variants, those that retain N-terminal ZFs but lack C-terminal ZFs (ZF5–6) are classified as dimerization-defective (DD) variants, as IKZF proteins rely on C-terminal ZFs for both homo- and heterodimerization. However, truncating variants may affect protein expression, alter DNA-binding specificity, interfere with posttranslational modifications such as SUMOylation, and disrupt broader protein–protein interactions beyond dimerization within the IKZF family. Recent studies revealed that the intrinsically disordered domain between ZF4 and ZF5 of IKAROS mediates interactions with the NuRD complex (48), whereas ZF2–3 interact with Foxp3 (10). Truncating variants lacking these domains, as well as missense variants in these regions, may disrupt IKAROS interactions with these molecules. A missense variant in the N terminus of IKAROS (D22N) has been shown to impair protein stability and functions as an HI variant (44). Missense variants affecting IKAROS-N159 are considered DN because the mutant protein loses its DNA-binding ability and interferes with the function of wild-type IKAROS, likely via homodimerization (23). Moreover, these variants may impair the DNA binding of other family members, such as AIOLOS, possibly through heterodimer formation.

### Clinical manifestation

Most patients with IKAROS deficiency develop the first signs of the disease by young adulthood (Fig. 1 C). However, several cases present during adulthood, indicating variable penetrance and a broad age of onset, which may reflect a progressive immune deficiency. Recurrent infections, particularly sinopulmonary bacterial infections, are common initial manifestations. Overall, infectious complications occurred in approximately half of all patients with IKAROS deficiency (Fig. 1 D). Up to 67% of the patients with hypoimmunoglobulinemia or B lymphopenia experience infectious complications. Causative microorganisms include bacteria, viruses, fungi, and parasites, which reflect the broad spectrum of infections observed in patients with IKAROS deficiency (reviewed in [49]). IKAROS-DN variants are associated with combined immunodeficiency, and all (9/9) infants with IKAROS-N159S/T variants experienced opportunistic infections such as PCP (21, 23, 43) (Fig. 1 A). In addition to PCP, patients with IKAROS-DN variants develop various bacterial, viral, and fungal infections.

Immune dysregulation is a common feature of IKAROS deficiency, occurring in ∼36% of the patients (Fig. 1, C and D). Autoimmune manifestations are frequent, with immune thrombocytopenia (ITP) and systemic lupus erythematosus (SLE) being the most commonly reported. Approximately 10% of patients experience chronic diarrhea, which is likely attributable to intestinal inflammation, although colonoscopic findings vary. Patients with IKAROS-DD variants presented phenotypes related to immune dysregulation (43%) rather than infections (30%).

Malignancy is an occasional complication of IKAROS deficiency (Fig. 1 D). Acute lymphoblastic leukemia (ALL) of both B and T cell origins has been reported. Among a cohort of 4,963 pediatric patients with ALL, 43 (0.9%) carried germline variants of the *IKZF1* gene, comprising 27 unique variants (50). It is likely that additional patients with ALL presenting with minimal or no overt immunodeficiency remain unrecognized. Other hematological malignancies, such as central nervous system lymphoma and Burkitt lymphoma, have occasionally been documented. Although *IKZF1*-N159S has been identified as a recurrent somatic hotspot mutation in acute myeloid leukemia (AML) (51), AML has not been reported in patients with IKAROS-DN variants in IEI cohorts.

One patient with IKAROS deficiency carrying a heterozygous IKAROS-C182X variant was identified through newborn T cell receptor excision circle (TREC) screening (34). A subset of patients exhibits a progressive decline in peripheral blood B cell counts over time. Conversely, spontaneous recovery from T and B lymphopenia, as well as pancytopenia, has been reported in a few other cases (21, 22, 43).

### Immunological features

Immunologically, IKAROS deficiency is characterized by variable degrees of B lymphopenia and hypoimmunoglobulinemia (Fig. 1 A). Overall, 60% of patients presented with B cell deficiency (defined as B cells ≤1% of lymphocytes and/or ≤20/μl, unless otherwise specified) or B lymphopenia (defined as B cells ≤5% of lymphocytes and/or ≤100/μl, unless otherwise specified), and 62% of patients presented with low to undetectable levels of immunoglobulins. Decreased memory B cell numbers are occasionally observed. Immunoglobulin levels are correlated with B cell counts, and agammaglobulinemia is frequently observed in patients with profound B cell deficiency. The severity of B cell lymphopenia and hypoimmunoglobulinemia reflects the overall severity of immunodeficiency in patients with IKAROS deficiency. Even in patients where immunoglobulin levels are preserved, vaccine-specific antibody responses are often impaired, indicating defective humoral immunity. When examined, bone marrow findings typically show a reduction in HSCs, CLPs, and B lineage progenitors, although increased CLP populations have occasionally been reported. T cell abnormalities are less consistent but frequently include elevated CD8^+^ T cell counts, with an inverted CD4/CD8 ratio.

Patients with IKAROS-DD variants exhibit milder B and T cell abnormalities than those with IKAROS-DN, LOF, or HI variants. Approximately 57% (13/23) of patients presented with B lymphopenia; however, none demonstrated B cell deficiency. Agammaglobulinemia is rare, and approximately half of patients present with hypoimmunoglobulinemia.

In contrast, most patients with IKAROS-DN variants exhibit B cell deficiency and agammaglobulinemia. Patients with combined immunodeficiency exhibit more pronounced T cell abnormalities, characterized by a marked expansion of the naïve T cell compartment and a consistent reduction in memory T cells. All major Th subsets, including Tregs, were significantly decreased. In vitro T cell activation assays demonstrated impaired proliferative responses, indicating a cell-intrinsic defect in T cell function in this patient population. Neutropenia and reduced eosinophil counts in peripheral blood were also commonly observed in patients with IKAROS-DN variants, suggesting that these variants are associated with defects in myeloid cell differentiation.

### Treatment and outcome

Immunoglobulin replacement therapy and prophylactic antibiotics remain the primary treatment options for IKAROS deficiency, particularly in patients with hypoimmunoglobulinemia and significant infectious complications. Caution is warranted for patients harboring IKAROS-DN variants, as they often present with combined immunodeficiency and severe infections. These patients typically require aggressive management, including intensive antibiotic therapy and immunoglobulin replacement. Given the high incidence of PCP in these patients, prophylactic measures should be implemented. For patients presenting with autoimmune manifestations, corticosteroids and other immunosuppressive agents are administered based on specific conditions. In one patient with the IKAROS-R162Q variant, treatment with the Janus kinase inhibitor filgotinib for colitis resulted in significant improvement in endoscopic findings (52).

Hematopoietic cell transplantation (HCT) has been performed in a few patients with IKAROS-LOF variants (20, 22, 26). One patient with the IKAROS-Y210C variant underwent HCT due to pancytopenia but succumbed to infectious complications and multiorgan failure (20). Another patient with a heterozygous whole-gene deletion of *IKZF1* received HCT for relapsed ALL from an HLA-matched sibling donor who was an asymptomatic carrier of the same deletion (22). The recipient remained healthy and discontinued immunoglobulin therapy 4 years after transplant. One patient harboring the IKAROS-C150R variant, who experienced severe and recurrent infections, underwent allogeneic HCT from an HLA-identical sibling donor (26). The transplantation resulted in full donor chimerism and resolution of immunodeficiency.

Spontaneous hematological recovery has also been observed. A second patient with the IKAROS-Y210C variant, who presented with B cell deficiency and pancytopenia, experienced spontaneous recovery of the phenotype (21). This highlights the disease course variability, with both spontaneous recovery and progressive deterioration. Therefore, indications for HCT in patients with IKAROS-LOF, HI, or DD variants should be carefully assessed on a case-by-case basis.

Another subgroup of patients who required HCT included those with IKAROS-DN variants. Five of the nine reported patients underwent HCT because of severe infectious complications (31, 43, 53). One patient died of complications related to a preexisting infection. Another case involved a patient with the IKAROS-N159S variant who received a CD34-enriched haploidentical bone marrow transplant for profound lymphopenia and hypoimmunoglobulinemia (43). Although the graft was rejected, the patient experienced spontaneous recovery of autologous T cells. However, the patient later developed T-ALL and required a second unrelated donor transplantation, which resulted in complete donor chimerism and favorable outcomes.

## IKAROS-gain-of-function (GOF) disease

IKAROS is one of the few genes for which LOF and GOF variants can cause IEI. IKAROS-GOF disease is primarily characterized by diverse forms of immune dysregulation, including autoimmunity, allergies, and plasma cell proliferation. To date, 31 patients from 10 families have been identified and reported in the literature (28, 29, 30, 54, 55, 56) (Fig. 1 A).

### Genetics and pathogenesis mechanism

Human IKAROS-GOF disease is caused by heterozygous missense variants of the *IKZF1* gene. IKAROS-R183H/C variants represent hotspot mutations, accounting for most cases in the cohort of patients with IKAROS-GOF disease. Two distinct mechanisms have been proposed to explain the effect of GOFs. The R183 residue is located at the −3 position relative to the start of the α-helix within the DNA-binding ZF3 (Fig. 1 B). This residue restricts the flexibility of ZF3, and its substitution with histidine or cysteine is thought to increase its conformational adaptability, thereby enhancing the stability of ZF3–DNA interactions (54). The current study confirmed that these variants exhibit stronger binding to canonical IKAROS-binding motifs at lower protein concentrations, accompanied by increased transcriptional activity. Another variant, IKAROS-T398M, alters the phosphorylation site, leading to impaired phosphorylation of threonine residue (55). Under physiological conditions, the DNA-binding activity of IKAROS is regulated by its phosphorylation status. During S phase, IKAROS is highly phosphorylated, and dephosphorylation enhances its DNA-binding affinity and restricts cell cycle progression (57). The threonine-to-methionine substitution in IKAROS-T398M leads to increased DNA binding and transcriptional activity during S phase, resulting in delayed G1–S phase transition and reduced cellular proliferation.

### Clinical manifestation

IKAROS-GOF disease presents a distinct clinical phenotype distinguishable from IKAROS deficiency (Fig. 1, A, C, and D). Immune dysregulation was observed in 81% (24/30) of reported individuals with IKAROS-GOF variants and was present in nearly all symptomatic patients, except for three patients whose only manifestation was malignancy or lymphoproliferation. Allergy is a hallmark feature in approximately half of patients with IKAROS-GOF disease, unlike IKAROS deficiency, where allergic manifestations are rare. Common allergic conditions include atopic dermatitis, allergic rhinitis, food allergies, and asthma. Additional immune dysregulation phenotypes included enterocolitis (three patients) and sarcoidosis (two patients).

Lymphoproliferation was common, and plasma cell cytosis, occasionally manifesting as IgG4-related disease, emerged as a characteristic finding, documented in 13/31 patients (Fig. 1 D). An additional four patients exhibited lymphadenopathy of unknown cause, potentially indicating undiagnosed plasma cell cytosis. Dacryoadenitis, diagnosed as necrobiotic xanthogranuloma in one case, was documented in seven patients (23% of the cohort) and was frequently accompanied by exophthalmos. In the cases that underwent biopsy, lymphoplasmacytic infiltrates with increased IgG4-positive cells were documented (30, 56). Two individuals carrying the IKAROS-R183H variant developed multiple myeloma in adulthood (29, 56), highlighting the need for long-term monitoring of patients with plasma cell cytosis. Other B cell malignancies and non-hematological cancers were also frequently observed. Six patients with lymphoma (Hodgkin’s lymphoma, anaplastic lymphoma kinase [ALK]-negative anaplastic large cell lymphoma, lymphoplasmacytic lymphoma, and diffuse large B cell lymphoma [DLBCL]) and three patients with non-hematological malignancies (prostate cancer, skin cancer, and renal cell carcinoma) were reported. Overall, 32% (10/31) of individuals developed hematological and/or non-hematological malignancies, suggesting a stronger association with malignancy in IKAROS-GOF disease than in IKAROS deficiency (Fig. 1, C and D). However, patients with IKAROS-GOF disease are generally older than those with IKAROS deficiency. Given this age difference, any association between IKAROS-GOF variants and non-hematological malignancies should be interpreted cautiously.

Infectious complications were less common in IKAROS-GOF disease, occurring in 11 of 31 patients (Fig. 1, A, C, and D). Among these, recurrent respiratory tract infections (RTIs) were the most frequent, affecting 9/11 symptomatic individuals. Severe bacterial infections have been documented only sporadically, nearly always in patients receiving intensive immunosuppression for immune dysregulation or lymphoma therapy. Other infections are rare, although human papillomavirus infection has been reported in two patients and severe coronavirus disease 2019 (COVID-19) in three. Unlike other IKAROS deficiencies, hematological manifestations are rare and limited to one patient with myelodysplastic syndrome.

### Immunological features

Total B cell counts were unaffected in patients with IKAROS-GOF disease; however, mildly decreased B cells or decreased memory B cell fractions were occasionally observed. Two patient with the IKAROS-R183C variant presented with severe B lymphopenia, reaching a B cell deficiency level. In contrast, a reduced fraction of plasmablasts in the peripheral blood is commonly observed in IKAROS-GOF disease. In vitro plasma cell differentiation from memory B cells is enhanced in patients with IKAROS-GOF disease (54).

In contrast to the predominant CD8^+^ T cells with an inverted CD4/CD8 ratio in IKAROS deficiency, the CD4^+^ T cell fraction is expanded in patients with IKAROS-GOF disease (54, 55). Additionally, T cells were occasionally skewed toward the memory phenotype, and an increased CD57^+^ senescent phenotype was noted. Moreover, CD4^+^ T cells from patients with IKAROS-GOF variants exhibited strong skewing toward Th2 cells both in vivo and in vitro, and circulating follicular helper T cells (Tfh) increased. The number of Tregs is markedly reduced in patients with IKAROS-GOF disease.

### Treatment and outcome

The management of IKAROS-GOF disease includes immunoglobulin replacement therapy in patients presenting with hypoimmunoglobulinemia and/or recurrent infections. Manifestations of immune dysregulation have been treated with agents such as rituximab, corticosteroids, and sirolimus. In one patient with the IKAROS-R183C variant who presented with colitis, extensive immunosuppressive therapy was only partially effective (29). This patient, along with another carrying the IKAROS-R183H variant who developed relapsing lymphoma, ultimately underwent allogeneic HCT and achieved complete donor chimerism with disease resolution (25, 29). Two additional patients, one with DLBCL and the other with lymphoblastic lymphoma, received autologous HCT as part of their treatment. One patient achieved complete remission, while the other responded well to HCT and adjunctive therapy with rituximab. However, both patients later developed lymphoproliferation and IgG4-related disease, highlighting an underlying predisposition to immune dysregulation and lymphoid malignancies.

Immunomodulatory imide drugs (IMiDs) such as lenalidomide and pomalidomide target cereblon E3 ubiquitin ligase to natural neosubstrates, including IKAROS and AIOLOS. These neosubstrates are ubiquitinated and undergo proteasomal degradation (58). Given their mechanism of inducing IKAROS degradation, IMiDs have potential as targeted therapies for IKAROS-GOF disease. In vitro studies have shown that lenalidomide normalizes T cell cytokine production and restores plasma cell differentiation (54). However, their clinical efficacy remains unclear. In one patient with the IKAROS-R183H variant, lenalidomide treatment resulted in a poor response, necessitating HCT (25). In another cohort of patients harboring the IKAROS-R183H variant, lenalidomide treatment was effective in controlling immune dysregulation, including manifestations such as alopecia areata and IgG4-related disease, in three patients (56). However, one patient developed neutropenia, necessitating temporal discontinuation of lenalidomide therapy. Further studies involving larger patient cohorts are required to clarify the therapeutic utility and safety of lenalidomide and other IMiDs for IKAROS-GOF disease.

## HELIOS deficiency

**Figure 2.**
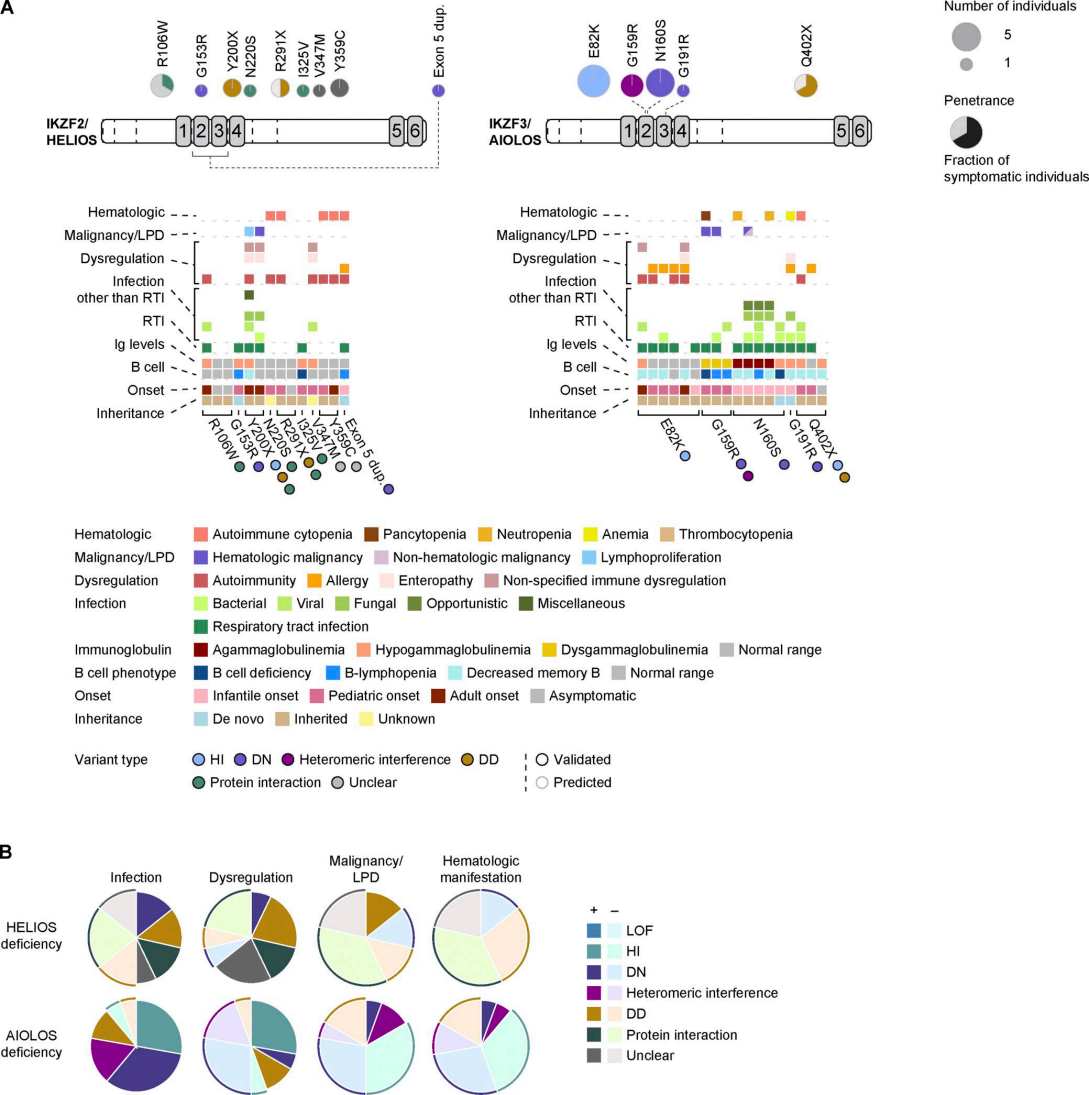
**HELIOS deficiency and AIOLOS deficiency. (A)** Structure of the IKZF2/HELIOS and IKZF3/AIOLOS is depicted, highlighting the N-terminal ZFs (ZF1–ZF4) responsible for DNA binding and the C-terminal ZFs (ZF5–ZF6) involved in homo- and heterodimerization, indicated by gray boxes. Exon boundaries within the structure are marked by dotted lines. Above, disease-causing variants associated with HELIOS and AIOLOS deficiencies are mapped, along with the number of individuals and the corresponding disease penetrance. Below, the clinical phenotypes reported by all known individuals with HELIOS and AIOLOS deficiency are shown. **(B)** Frequencies of symptomatic individuals for each phenotype are summarized for HELIOS and AIOLOS deficiencies. Color codes represent variant types.

HELIOS deficiency was first reported in 2021 as an IEI characterized mainly by autoimmune manifestations, enteropathy, and additional phenotypes associated with immune dysregulation. This deficiency results from either monoallelic or biallelic LOF variants in the *IKZF2* gene (59, 60, 61). HELIOS-I325V was reported to be biallelic and is the only autosomal recessive IKZF-associated IEI (60). Following these initial reports, heterozygous LOF variants in the *IKZF2* gene have been identified in patients with syndromes associated with immune dysregulation, low TRECs, craniofacial anomalies, hearing loss, and developmental delay, termed immunodysregulation, craniofacial anomalies, hearing impairment, athelia, and developmental delay (ICHAD) syndrome (62, 63). Recently, germline *IKZF2* variants were identified in a cohort of patients with non-syndromic hereditary hearing impairments (64). To date, 14 patients from nine families, each carrying a distinct variant, have been reported in the literature (59, 60, 61, 62) (Fig. 2 A).

### Genetics and pathogenesis mechanism

In contrast to other IKZF-associated IEI, most HELIOS deficiency–causing missense variants demonstrate normal DNA-binding capacity, as assessed using an electrophoretic mobility shift assay (EMSA) and/or pericentromeric heterochromatin (PC-HC) assays. As expected, the truncated variants exhibited impaired heterodimerization and homodimerization. The truncated HELIOS-Y200X protein was not detected in primary cells from affected patients, suggesting that this variant may function via an HI mechanism. Both missense (I325V, R106W, N220S) and truncating variants (Y200X, R291X) are associated with reduced interactions with NuRD complex components, such as histone deacetylase 1 (59, 60, 61). These findings support the hypothesis that impaired transcription factor complex formation and deficient recruitment of transcriptional regulatory machinery represent the central mechanisms of HELIOS deficiency. HELIOS-G153R and exon 5 duplication variants are considered DN, although the current data do not exclude LOF or HI mechanisms without DN effects (62). However, given that the homologous variant of AIOLOS (AIOLOS-G159R) demonstrates a DN mechanism, it is probable that HELIOS-G153R similarly acts via a DN mechanism (Fig. 1 B). The HELIOS-Y359C and V347M variants appear functionally neutral with respect to DNA binding and protein–protein interactions (61). Although the clinical phenotypes of these patients were consistent with HELIOS deficiency, the pathogenicity and underlying mechanisms of these variants remain unclear.

### Clinical manifestation

The clinical phenotypes of HELIOS deficiency are characterized by a high frequency of immune dysregulation, observed in approximately two thirds of the patients (9/14) (Fig. 2, A and B). Disease onset ranges from infancy to adulthood, with immune dysregulation phenotypes most commonly emerging from adolescence to young adulthood. ITP occurs in approximately one third of the patients with HELIOS deficiency. Other common autoimmune complications included autoimmune hemolytic anemia and SLE, each reported in two patients. Enteropathy was noted in 3/14 patients, although it was not pathologically confirmed to be immune-related. Allergy is rare; however, atopic dermatitis was identified in one patient harboring an exon 5 duplication.

Infectious complications, predominantly recurrent RTI, were observed in half of the patients reported with HELIOS deficiency (Fig. 2, A and B). In approximately two thirds of these patients, the infections begin during adulthood, suggesting a relatively mild immunodeficiency. However, one patient carrying the HELIOS-V347M variant developed chronic active Epstein-Barr virus (EBV) infection (CAEBV) and EBV-related hemophagocytic lymphohistiocytosis (EBV-HLH). Another patient with the HELIOS-R106W variant experienced infectious mononucleosis (IM) and virus-associated hemophagocytic syndrome, although the causative pathogen was not described. Oral and vaginal candidiasis have been documented in patients with the HELIOS-Y200X variant.

Lymphoproliferative disorders and lymphoid malignancies occasionally occur, similar to other IKZF-associated IEI (Fig. 2, A and B). Two family members with the HELIOS-Y200X variant developed splenomegaly, and one subsequently progressed to Hodgkin’s lymphoma. Unlike other IKZF-associated IEI, hematological manifestations such as cytopenia or other hematological abnormalities have not been reported in patients with HELIOS deficiency, apart from autoimmune cytopenia.

Additionally, hypothyroidism was observed in three patients, although its association with HELIOS deficiency remains unclear. Given the identification of germline heterozygous *IKZF2* variants (N35S and L328V) in patients with autoimmune endocrine disease, although functionally unvalidated (65), hypothyroidism may be autoimmune in origin.

### Immunological features

As observed in other IKZF-associated IEI, patients with HELIOS deficiency exhibited variable degrees of B cell abnormalities (Fig. 2 A). B cell deficiency was explicitly observed in patients with the homozygous HELIOS-I325V variant, while B lymphopenia was noted in patients with HELIOS-DN variants. A reduction in memory B cells has been detected in other subsets of patients. Hypoimmunoglobulinemia was associated with individuals who experienced recurrent infections; however, none of the patients had agammaglobulinemia. Reduced natural killer cells, inverted CD4/CD8 ratio, and reduced circulating Tfh levels are among the frequently observed immunological abnormalities. Both CD4^+^ and CD8^+^ T cells exhibited a skewing toward memory phenotypes. Low TREC levels and T lymphopenia are characteristics of patients with HELIOS-DN. Although Tregs were present, occasional mild reductions in their numbers were noted (HELIOS-I325V and Y200X variants). Lymphadenectomy was performed in patients with the HELIOS-Y200X variant who presented with lymphadenopathy. Histological analysis revealed an accumulation of Tfh-like cells in the light zones of the lymph nodes, indicative of a dysregulated germinal center reaction (59). Mucosal-associated invariant T cells were reduced in the peripheral blood of the patients who were tested (HELIOS-I325V and Y200X variants).

### Treatment and outcomes

Prophylactic antibiotics and/or immunoglobulin replacement therapy are administered to patients with significant infectious complications. For immune dysregulation manifestations, such as SLE, systemic immunosuppressants, including corticosteroids and hydroxychloroquine, have been used with satisfactory clinical responses. In one patient carrying the HELIOS-V347M variant who developed CAEBV and EBV-HLH, HCT was performed following the failure of multiple immunomodulatory therapies (61, 66). HCT achieved complete donor chimerism and successfully cured both CAEBV and EBV-HLH; however, the patient subsequently developed an autologous EBV-positive T cell lymphoma (66). To date, no fatal complications have been reported in the affected individuals.

## AIOLOS deficiency

The first reports of AIOLOS deficiency described two missense variants, one of which (AIOLOS-N160S) is homologous to a previously identified IKAROS-DN variant (IKAROS-N159S). Patients with the AIOLOS-N160S variant exhibited phenotypes similar to those seen in patients with IKAROS-DN variants, including PCP, B cell deficiency, and agammaglobulinemia (67). Another variant, AIOLOS-G159R, is associated with immunodeficiency complicated by EBV-associated B cell lymphoma (68). AIOLOS-G159R was the first IKZF-associated IEI for which a knock-in mouse model was used to confirm the pathogenicity of the variant and elucidate its molecular mechanism. As observed in IKAROS deficiency, both HI variants and variants with DD have been identified in AIOLOS deficiency (69). To date, 18 patients harboring five distinct *IKZF3* variants have been reported in the literature (67, 68, 69, 70, 71) (reviewed in [72]) (Fig. 2 A).

### Genetics and pathogenesis mechanism

Like IKAROS deficiency, most missense variants causing AIOLOS deficiency (G159R, N160S, and G191R) are located within DNA-binding ZFs (Fig. 1 B). These variants are located at positions 2, 3, and 6 relative to the start of the α-helix within the ZF domain. These residues are located at the DNA-binding interface and are critical in mediating DNA interactions. Both AIOLOS-N160S and G191R function as DN variants, with mutant proteins interfering with the DNA binding of wild-type AIOLOS, as demonstrated using EMSA and PC-HC assays (67, 70). Although IKAROS-N159S (the homologous variant of AIOLOS-N160S) impaired the DNA-binding ability of its heteromeric partner AIOLOS, AIOLOS-N160S did not suppress IKAROS DNA binding. Moreover, the AIOLOS-N160T substitution (homologous to IKAROS-N159T, another IKAROS-DN variant) is considered a benign polymorphism given its allele frequency of ∼1/300 in the general Korean population (rs1598018757 in single nucleotide polymorphism database [dbSNP]). In contrast, AIOLOS-G159R loses its DNA-binding capacity and interferes with the DNA binding of IKAROS, a mechanism referred to as heteromeric or heterodimeric interference (68). Furthermore, AIOLOS-G159R acquires a new binding affinity for noncanonical sequences. The molecular basis of these differences, as well as their specific consequences on immune function, remains unclear and warrants further investigation.

The AIOLOS-E82K variant, located in the N-terminal region, is thought to affect protein stability and acts via an HI mechanism (69). However, given that stop-gain variants in the *IKZF3* gene are not tolerated in the general population (probability of being LOF-intolerant score = 1, sourced from gnomAD) and considering the relatively high frequency of AIOLOS-E82K (minor allele frequency 1.53e–4, sourced from gnomAD), this variant may not represent classical HI and could allow for residual protein expression. The AIOLOS-Q402X variant lacks C-terminal ZFs and is expected to behave as a DD variant. However, it compromises protein stability and is likely to act, at least in part, through the HI mechanism. Studies on AIOLOS-DD variants have revealed that heteromerization with AIOLOS can be mediated through the N-terminal ZF1 without C-terminal ZFs.

### Clinical manifestation

Recurrent sinopulmonary infections were the most common infectious complications of AIOLOS deficiency, observed in most patients (15/18) (Fig. 2, A and B). Various bacterial and viral infections have been reported (as detailed in [72]), whereas fungal infections are rare, except for PCP, which occurs in patients with the AIOLOS-N160S variant. Similar to IKAROS-DN variants, the AIOLOS-DN variant is associated with a combined immunodeficiency phenotype. Half of the patients carrying AIOLOS-DN variants developed PCP. These patients experienced other opportunistic infections, including *Pseudomonas* sepsis or pneumonia, and meningitis of unknown etiology.

However, the severity of immunodeficiency in patients with the AIOLOS-G159R variant appears to be milder than that observed in patients with AIOLOS-DN variants. Although recurrent RTIs are common (2/3), fungal or other opportunistic infections have not been reported. One patient with the AIOLOS-G159R variant developed recurrent IM due to EBV and subsequently developed CAEBV, a phenotype reminiscent of that of patients with the HELIOS-V347M variant. The patient died of gastrointestinal bleeding secondary to CAEBV. Patients with AIOLOS-DD or HI variants frequently experienced recurrent RTIs (7/9) and occasional bacterial infections.

Immune dysregulation is a common clinical feature among patients with AIOLOS-HI variants, affecting 7/9 individuals, with onset ranging from childhood to adulthood (Fig. 2, A and B). Overall, immune dysregulation–related manifestations were observed in approximately half of all the patients reported with AIOLOS deficiency (8/18). In contrast to patients with AIOLOS-HI variants, those with AIOLOS-DN and AIOLOS-G159R variants rarely exhibited immune dysregulation. One patient with the AIOLOS-G191R variant presented with a food allergy. Like other IKZF-associated IEI, autoimmune conditions such as ITP and SLE were reported in five out of nine patients with AIOLOS-HI variants. Among them, Hashimoto’s thyroiditis was observed in three patients. Allergic manifestations, including bronchial asthma (3/9), atopic dermatitis (2/9), and food allergy (1/9), were common, affecting 5/9 patients with AIOLOS-HI variants. One patient with the AIOLOS-E82K variant developed colitis.

Malignancies have occasionally been reported in AIOLOS deficiency, with an overall frequency of 3 of 18 patients (Fig. 2, A and B). Hematological malignancies included EBV-positive B cell lymphoma (EBV-positive DLBCL, with or without follicular lymphoma), which was reported in 2/3 of patients with the AIOLOS-G159R variant. Additionally, one of the five patients with the AIOLOS-N160S variant developed chronic lymphocytic leukemia and subsequently metastatic melanoma.

Lymphopenia and anemia were observed in patients with the AIOLOS-G191R variant, whereas pancytopenia was reported in one of three patients with the AIOLOS-G159R variant.

### Immunological phenotype

B cell developmental defects are the most prominent immunological abnormalities associated with AIOLOS deficiency. These defects ranged from B cell deficiency (observed in 1/3 of patients with the AIOLOS-G159R variant and 1/5 with the AIOLOS-N160S variant) to B lymphopenia (2/3 of AIOLOS-G159R and 1/5 of AIOLOS-N160S) and reduced memory B cell fractions (seen in most patients with AIOLOS-DN and HI variants) (Fig. 2 A). Altogether, B cell developmental defects were identified in 16 of the 18 patients with AIOLOS deficiency. Despite B cell deficiency or B lymphopenia, patients with the AIOLOS-G159R variant frequently exhibit dysgammaglobulinemia, characterized by elevated levels of IgG, IgE, or IgA. Most (4/5) of the patients with the AIOLOS-N160S variant were agammaglobulinemic, similar to those with the IKAROS-N159S variant. The remaining patients with AIOLOS-DN variants exhibited hypoimmunoglobulinemia. Moreover, B cells from patients with the AIOLOS-N160S variant demonstrated impaired differentiation into memory B cells and plasmablasts upon stimulation with CD40L and IL-21. In AIOLOS-HI variants, hypoimmunoglobulinemia was observed in approximately half of the patients (4/9).

T cell abnormalities were more variable in AIOLOS-deficient individuals. In patients with the AIOLOS-G159R variant, CD4^+^ T cells displayed a skewing toward a memory phenotype, with imbalanced helper T cell subsets, and decreased Th17 and increased Th1* (defined by CD3^+^CD4^+^CD45RO^+^CD161^+^CCR6^+^CXCR3^+^) cells. In patients with the AIOLOS-N160S variant, reductions in memory T, Th1, and Tfh cells were commonly observed. Impaired induction of CD40L expression upon T cell activation was observed. In a patient harboring the AIOLOS-N160S variant, a reduced fraction of CD62L^+^ T cells was observed, a phenotype that was recapitulated in the corresponding knock-in mouse model (71, 73). Further studies involving more patients are required to confirm these findings. T lymphopenia and reduced naïve T cell numbers are occasionally observed in patients with AIOLOS-HI and AIOLOS-G191R variants.

### Treatment and outcomes

Similar to other IKZF family-associated IEI, immunoglobulin replacement therapy has been used in patients with AIOLOS deficiency who present with hypoimmunoglobulinemia and/or severe infectious complications. Given the high incidence of PCP in individuals with the AIOLOS-N160S variant, PCP prophylaxis should be considered in this group of patients.

In patients with AIOLOS-HI/DD variants, immune dysregulation has been managed by various immunosuppressive agents, including corticosteroids, mycophenolate mofetil, hydroxychloroquine, methotrexate, and omalizumab. The clinical responses varied among the patients.

Allogeneic HCT was performed in two patients with the AIOLOS-G159R variant, both of whom presented with lymphoma. While HCT was pursued as a curative approach for malignancy and immune abnormalities associated with AIOLOS deficiency, one of the two patients died of transplant-related complications.

## Conclusion

**Figure 3.**
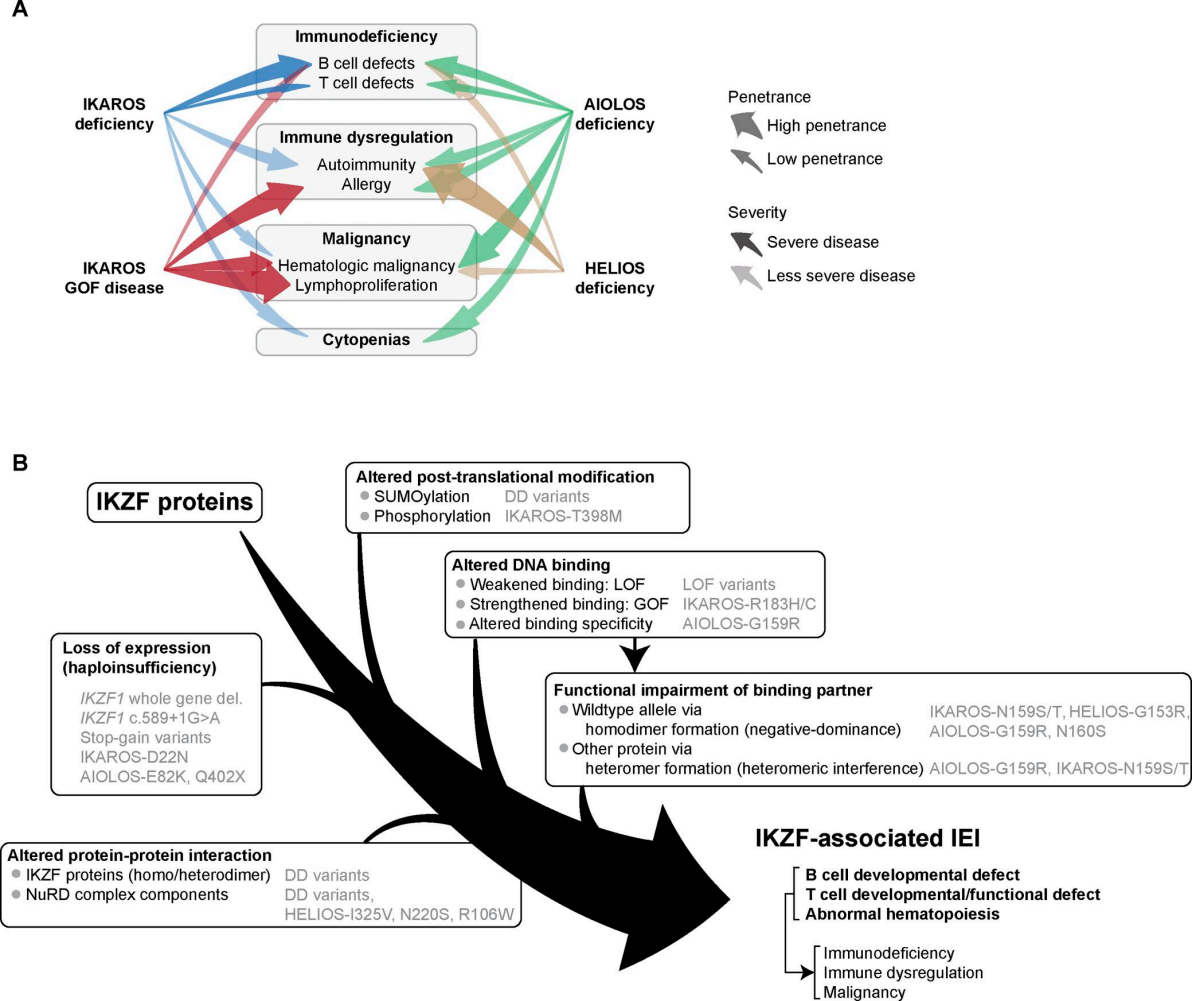
**Clinical summary and molecular pathogenesis of IKZF-associated IEI. (A)** Summary of immune abnormalities and associated phenotypes in IKZF-associated IEI. Arrow width indicates penetrance, while color intensity reflects the severity of each phenotype. **(B)** Molecular mechanisms underlying disease pathogenesis for IKZF-associated IEI, along with the associated variants, are illustrated.

IKZF transcription factors play crucial roles in the differentiation and function of hematopoietic cells. Germline variants of *IKZF1*, *IKZF2*, and *IKZF3* cause IKZF-associated IEI (Fig. 3 A; and Tables 1 and S1). IKAROS deficiency leads to B cell and antibody defects with variable T cell dysfunction and a spectrum of immune dysregulation, including autoimmunity and occasional malignancies. IKAROS-GOF disease predominantly presents with immune dysregulation, including allergy, autoimmunity, and lymphoproliferation, with plasma cell expansion. HELIOS deficiency predominantly presents with autoimmune diseases and is occasionally accompanied by infections and syndromic features, including developmental delay and hearing loss. AIOLOS deficiency manifests as an antibody deficiency and variable T cell abnormalities, and HI variants are more strongly linked to autoimmunity and allergies.

**Table 1.** Summary of clinical and immunological phenotypes of IKZF-associated IEI

​	IKAROS deficiency	IKAROS-GOF disease	HELIOS deficiency	AIOLOS deficiency
Mechanisms	LOF, HI, DD, DN	GOF	LOF, altered protein interaction, DD	LOF, DN, heteromeric interference, HI, DD
Causative gene	*IKZF1*	*IKZF1*	*IKZF2*	*IKZF3*
Inheritance	AD	AD	AD or AR	AD
Reported frequency	1–2% to 10% within CVID cohort (45, 46, 47)	Unknown	Unknown	Unknown
Immunological abnormalities				
B cell phenotype	B cell deficiency ∼ B lymphopenia (often progressive, mild in DD)	Occasional B lymphopenia	Occasional B lymphopenia	↓ memory B, occasional B lymphopenia
Immunoglobulin levels	Agammaglobulinemia ∼ hypoimmunoglobulinemia	Dysgammaglobulinemia	Occasional hypoimmunoglobulinemia	Agammaglobulinemia ∼ normal range
T cell phenotype	Variable, ↓ memory T in DN	↑ CD4^+^ T_EM_ and CD8 T_EMRA_, Th2 skewing, ↓ Treg	CD4^+^ T and CD8^+^ T skewing toward memory. Occasional ↓ number or ↓ suppressive function of Treg	Variable, ↓ naïve CD4^+^ T (other than DN), occasional CD4^+^ T skewing toward naïve in DN
Clinical manifestations				
Infections	++ (mild in DD), +++ (DN)	+	++	++, +++ (DN)
Immune dysregulation	+	++	++	++ (HI and DD)
Malignancy	+	+++	+	+
Cytopenias	+	− (occasional AIC)	− (frequent AIC)	+
Syndromic manifestation	−	−	+ (ICHAD syndrome)	−

+, occasional; ++, frequent; +++, frequent and severe. AIC, autoimmune cytopenia; AD, autosomal dominant; AR, autosomal recessive; CID, combined immunodeficiency; T_EM_, effector memory T cells; T_EMRA_, effector memory T cells re-expressing CD45RA.

At the molecular level, pathogenic LOF variants in IKZF-associated IEI have been shown to disrupt protein function through one or a combination of the following mechanisms: (1) loss of expression of the mutant allele, leading to HI; (2) defective DNA binding; (3) altered protein interactions, including impaired homo- and heterodimerization, as well as other protein–protein interactions; and (4) changes in posttranslational modifications that affect protein function (Fig. 3 B). In contrast, GOF variants in IKAROS-GOF disease enhance the DNA binding and transcriptional activity of the protein by altering the flexibility of DNA-binding ZF or by losing the phosphorylation residue.

Emerging evidence suggests genotype–phenotype correlations, even across different IKZF genes. For example, patients with IKAROS-N159S and AIOLOS-N160S variants and homologous mutations in their respective DNA-binding ZFs exhibited similar combined immunodeficiency phenotypes. Additionally, variants predicted to impair dimerization in both IKAROS and AIOLOS were relatively enriched in patients with immune dysregulation–associated phenotypes.

However, these experimentally defined mechanisms of action do not align consistently with the clinical outcomes. Patients harboring the same or mechanistically similar variants often present a broad spectrum of phenotypes. However, the causes underlying this phenotypic variability are poorly understood. Multiple functional disruptions, which frequently occur in combination, contribute synergistically to the complex immune dysregulation observed in IKZF-associated disorders (Fig. 3 B). Additionally, expression of affected genes varies according to the cell type and differentiation stage. Moreover, allelic bias, such as monoallelic expression, may lead to cellular “mosaicism” in patients carrying germline variants, potentially contributing to complex traits with incomplete penetrance (74). For example, in IKZF-associated IEI caused by haploinsufficient variants, the monoallelic expression of the affected allele can result in complete loss of protein expression, whereas expression of the wild-type allele yields normal protein levels. When such monoallelic expression occurs in a lineage- or stage-specific manner during hematopoietic differentiation, it may lead to selective deficits in certain cell populations. This cellular mosaicism may, in turn, contribute to the phenotypic heterogeneity observed among affected individuals.

Accumulating evidence indicates that IKZF-associated IEI present with a broad spectrum of clinical and immunological phenotypes, even among patients harboring the same genetic variant. Therefore, genetic testing plays a critical role in establishing the diagnosis. Notably, many pathogenic variants associated with IKZF-related IEI exhibit incomplete penetrance, and some are observed at relatively high allele frequency in the general population. Therefore, functional analyses are essential to assess the pathogenic potential of these variants.

The management of IKZF-associated IEI remains largely symptomatic. Current approaches include prophylactic antimicrobials and/or immunoglobulin replacement therapy in patients with infectious complications or hypoimmunoglobulinemia. Immunosuppressive agents tailored for specific clinical conditions are used to manage immune dysregulation.

For patients with severe disease that manifests as recurrent infections, profound immune dysregulation, or malignancy, allogeneic HCT is currently considered the only curative option. Although HCT has led to successful outcomes in most cases, transplant-related mortality can occur, particularly in patients with significant comorbidities. Donor selection requires careful consideration, especially when related donors are used, because asymptomatic individuals may harbor pathogenic variants. Moreover, because spontaneous improvement and progressive immune decline have been reported in IKZF-associated IEI, the indication for HCT should be based on a comprehensive assessment of disease severity and course.

Advances in our understanding of molecular pathogenesis have paved the way for molecular-targeted therapies. For instance, lenalidomide has been shown to reverse T and B cell immune abnormalities in IKAROS-GOF disease in vitro, suggesting the potential utility of IMiDs in IKAROS-GOF disease. The clinical efficacy of lenalidomide has been documented in several cases. Existing IMiDs lack specificity, targeting IKAROS, AIOLOS, and other ZF proteins. The development of proteolysis-targeting chimeras with improved substrate specificity would allow for the selective degradation of mutant IKAROS proteins while sparing wild-type and related ZF proteins. Additionally, bortezomib, a proteasome inhibitor, has been shown to restore AIOLOS-E82K (a variant affecting the stability of AIOLOS, acting as an HI) in vitro in cell lines. Although further validation using patient-derived cells is required, proteasome inhibitors may represent a potential strategy for treating missense variants that impair protein stability and act via an HI mechanism. However, such variants may also confer additional deleterious effects on protein function. Therefore, comprehensive molecular characterization of each variant is essential to fully assess its pathogenic mechanism and therapeutic potential. In a murine model, genetic deletion of the C-terminal ZFs in Aiolos-G158R mice (a knock-in model of AIOLOS-G159R) abolished the interaction between mutant Aiolos and Ikaros, thereby eliminating heteromeric interference (68). This intervention rescued B cell development, suggesting that disrupting AIOLOS dimerization may represent a potential therapeutic strategy for variants acting through a heteromeric interference mechanism, by preventing their DN effects on other IKZF family proteins.

For IKZF-associated IEI, where the central pathogenesis involves significant cell-intrinsic differentiation defects, gene correction therapy holds promise as a potentially curative therapeutic approach. However, beyond addressing the immunodeficient phenotype, it is crucial to evaluate whether gene correction can prevent the development of autoimmunity and hematological malignancies in the long term. This is important, given the possibility that residual host cells may retain their intrinsic pathogenic potential.

Since the first report of IKAROS deficiency in 2012, the number of documented cases of IKZF-associated IEI has exceeded 180. The field of IKZF-associated IEI continues to expand, as exemplified by the recent identification of IKAROS-GOF disease and the extension of causative genes, including *IKZF2* and *IKZF3*. Emerging disease entities, such as ICHAD syndrome, associated with *IKZF2* variants, suggest that additional phenotypes and genotypes are yet to be identified. As awareness increases and molecular diagnostics improve, more patients will likely be identified, further broadening the clinical spectrum. Continued progress in molecular and clinical immunology is essential to deepen our understanding of these complex disorders and drive the development of curative therapies that can significantly improve patient outcomes.

## Online supplemental material

Table S1 summarizes the categories of IKZF-associated IEI as classified in the 2024 International Union of Immunological Societies (IUIS) classification for IEI (75), along with their clinical characteristics in comparison with other disorders within the corresponding IUIS categories.

## Supplementary Material

Table S1 shows IKZF-associated IEI in the 2024 IUIS classification and their characteristic clinical manifestations within each disease category.
