# Supplementary material for: IKZF-associated inborn errors of immunity
Source: J Hum Immun. 2025 Jul 18;1(3):e20250063. doi: 10.70962/jhi.20250063 (PMC13177390; doi:10.70962/jhi.20250063)
Supplement: Table S1 — shows IKZF-associated IEI in the 2024 IUIS classification and their characteristic clinical manifestations within each disease category. [file jhi_20250063_tables1.docx]

**Table S1.** IKZF-associated IEI in the 2024 IUIS classification and their characteristic clinical manifestations within each disease category.

|  | **IKAROS deficiency** | **IKAROS-GOF disease** | **HELIOS deficiency** | **AIOLOS deficiency** |
| --- | --- | --- | --- | --- |
| **IUIS classification(1)** | Predominantly antibody deficiency (LOF, HI, DD); Immunodeficiencies affecting cellular and humoral immunity (DN) | Diseases of immune dysregulation | Immunodeficiencies affecting cellular and humoral immunity | CIDs with associated of syndromic features |
| **Characteristic manifestation*** | Complications of immune dysregulation, hematological malignancies, and cytopenias (LOF, HI, DD); B cell deficiency and agammaglobulinemia (DN) | Plasma cell cytosis and IgG4-related disease | High incidence of autoimmune complications and syndromic features associated with specific variants | ** |

* Distinct clinical manifestations in comparison to other diseases within their respective IUIS classification groups.

** rarity of reported cases limits detailed phenotypic characterization among the IEI within the category. AIOLOS deficiency should be considered in the differential diagnosis of IKAROS deficiency due to overlapping clinical features.

**Reference**

1. Poli, M.C., I. Aksentijevich, A.A. Bousfiha, C. Cunningham-Rundles, S. Hambleton, C. Klein, T. Morio, C. Picard, A. Puel, N. Rezaei, et al. 2025. Human inborn errors of immunity: 2024 update on the classification from the International Union of Immunological Societies Expert Committee. *Journal of Human Immunity* 1:10.70962/jhi.20250003
